# Power mean based image segmentation in the presence of noise

**DOI:** 10.1038/s41598-022-25250-x

**Published:** 2022-12-07

**Authors:** Afzal Rahman, Haider Ali, Noor Badshah, Muhammad Zakarya, Hameed Hussain, Izaz Ur Rahman, Aftab Ahmed, Muhammad Haleem

**Affiliations:** 1 Department of Mathematics, University of Peshawar, Peshawar, Pakistan; 2 Department of Basic Sciences, University of Engineering and Technology Peshawar, Peshawar, Pakistan; 3 Department of Computer Science, Abdul Wali Khan University, Mardan, Pakistan; 4 Department of Computer Science, University of Buner, Buner, Pakistan; 5 Department of Computer Science, Kardan University, Kabul, Afghanistan

**Keywords:** Computer science, Image processing

## Abstract

In image segmentation and in general in image processing, noise and outliers distort contained information posing in this way a great challenge for accurate image segmentation results. To ensure a correct image segmentation in presence of noise and outliers, it is necessary to identify the outliers and isolate them during a denoising pre-processing or impose suitable constraints into a segmentation framework. In this paper, we impose suitable removing outliers constraints supported by a well-designed theory in a variational framework for accurate image segmentation. We investigate a novel approach based on the power mean function equipped with a well established theoretical base. The power mean function has the capability to distinguishes between true image pixels and outliers and, therefore, is robust against outliers. To deploy the novel image data term and to guaranteed unique segmentation results, a fuzzy-membership function is employed in the proposed energy functional. Based on qualitative and quantitative extensive analysis on various standard data sets, it has been observed that the proposed model works well in images having multi-objects with high noise and in images with intensity inhomogeneity in contrast with the latest and state-of-the-art models.

## Introduction

Image segmentation is a fundamental step in computer vision and in digital image processing. The main idea of image segmentation is to visualize meaningful objects in a given scene or image^1,2^ linked to many important fields such as medical imaging, object detection, video, traffic control systems, surveillance, automated surgeries, and so on^3–5^. Several state-of-the-art approaches for image segmentation exist; and some well-known methods include clustering^3^, thresholding^6^, edge detection and region-based models^7–10^, Markov random fields^7,8^, and stochastic methods^9,10^, etc. However, images are diverse in nature, and frequently happens that one model working for a particular class or type of images but may not properly work for other types. Some well-known factors which normally affect the performance of the segmentation models are noise and intensity in-homogeneity in a given image^4,11^. To cope with these issues, and to achieve accurate image segmentation, the active contour framework is a very popular technique due to its flexibility of allowing and imposing the desired constraints. Moreover, the availability of robust implementations, such as efficient optimization, and fast numerical methods are crucial. The main idea of active contour methods, as suggested in^1,7–10,12–17^, is to allow dynamical curves to move autonomously on a given image which, in fact, locates boundaries of the objects/regions therein.

The active contour models mainly use the concept of variational calculus^1,21–25^, that is functional optimization. It can be easily observed that the variational models for image processing in general and for segmentation, in particular, derive an energy functional which is minimized to get the desired results. The active contour models can be mainly divided into three categories, region-based^1,12–16,18^, edge-based models^7–10^ and region and edge based models^26^. A milestone variational model for segmentation purposes introduced by Mumford-Shah (MS )^1^ aims to obtain a smooth cartoon image that leads to edge detection. It is important to note that the design of the MS model is for ideal images, that is, images without noise, outliers and intensity in-homogeneity. Moreover, the direct implementation of this model is not feasible^22^. To easily implement the MS model, Chan *et al.* (CV)^9^ restricted the MS model to a piece-wise function reconstruction (two phases). By phase here we mean the set of homogeneous intensity pixels which can be easily distinguished from other sets of homogeneous intensity pixels in a given image. We should emphasize that the CV model ignores the presence of noise and other factors such as intensity in-homogeneity^23^. To improve the CV model, many techniques has been introduced in the last decades. In contrast with CV model, Li *et al.*^39^ proposed the Local Binary Fitting (LBF) model which performs much better and resembles than the MS model by carrying out the segmentation via approximating given image with two locally smooth functions.

Through balancing the local and global information, Mondal *et al.*^16^ pioneered a revolutionary methodology in their work. The model performs exceptionally well for images, in fact, with noise, inhomogeneity in intensity, and it happens within the presence of outliers. According to numerous experimental and numerical outcomes that we observed for various datasets, the model can successfully handle all images with: (i) intensity inhomogeneity, (ii) fuzzy border or discontinuous margins, and (iii) the presence of moderate noise. In addition, both Chuang *et al.*^19^ and Tripathy *et al.*^20^ provided models that are claimed to perform better for MRI images that are, in particular, noisy; but these models may perform worse or less well for those images that have greater impacts of intensity inhomogeneity. Since, these anticipated models are not convex, therefore it is essential and required to make multiple adjustments and considerable modifications to the original guess’s position in order to get the desired outcomes, and results. The concept of Coefficient of Variation (CoV) is the foundation of the model that is demonstrated in Wu *et al.*^24^ which is, in fact, a convex variational segmentation model and has received more attention in the image segmentation literature. This should be noted that this particular approach overlooks the factor and presence of noise and outliers in given images. In fact, the authors provide evidence for this assertion using the CoV-based image data fitting term, which is the sum of squares divided by the total of image intensity.

Similar to the average fitting term in the CV model, the value of the average fitting term in the Wu *et al.*^24^ model shows sensitivity to noise and outliers^27^. To further improve this model, Wu *et al.*^12^ proposed an active contour model incorporating a kernel metric, which is robust, stable, and works well for images with low noise and outliers. Ali *et al.*^28^ introduced the Lehmer’s type generalized mean which is mathematically expressed as given in Eq. (1).

1 $$\begin{aligned} \frac{\sum _{i,j}{\textbf{u}(i,j)}^p}{\displaystyle \sum _{i,j}{\textbf{u}(i,j)^{p-1}}}, \end{aligned}$$

Note that the above formula shows the Lehmer’s type generalized mean in a segmentation framework, where *p* is any real number. Although, this average is very effective in multi-region segmentation and suitable to different image intensity backgrounds, however it requires further analysis to tackle noise and outliers.

Chan *et al.*^9^ restricted the MS model to a piece-wise function reconstruction, but due to non-convexity, one must tune several times the position of the initial guess for the desired results. Krinidis *et al.*^31^ proposed a fuzzy energy-based active contour model, but it may not perform well for noisy images because of the old conventional least square objective function. Wu *et al.*^12^, proposed a fuzzy active contour model which gets enough weights to affect the segmentation performance in noisy images. The results of Li *et al.*^15^ is less efficient for noisy images. Wu *et al.*^24^ proposed a strictly convex model, but their objective function is sensitive to noise and outliers^27^. As compared to^12,15,27,31^ models a new objective function is used and the results clearly depicts that our work out performs.

From the above discussion, we can observe that most of the variational region-based image segmentation models, in the existing literature, are based on the least square function. In fact, this forces the fit of the data to a piece-wise function of the mean intensity values of the foreground and background. Moreover, they are unable to fully discriminate the noise and intrinsic intensities in the images. This is one the main reasons that the aforementioned models and similar frameworks are unable to correctly and appropriately segment noisy and outliers affected images^28^.

In this article, we mainly focus to design an efficient image data fitting term based on a novel objective function, as given by Eq. (2).

2 $$\begin{aligned} \displaystyle \left( \frac{1}{\mid \Omega \mid }\sum _{i,j}(||\textbf{u}(i,j)-a||_{2}^{2})^{p}\right) ^{\frac{1}{p}}. \end{aligned}$$

As further will be explained in “Proposed model” section, this term is robust against the outliers by giving very fewer weights to outliers and noise in contrast compare with the traditional and old objective function which gives equal or almost equal weights to outliers and true image pixels^29^. Moreover, besides the new data fitting term of the proposed model, a fuzzy level set function is employed which has two main benefits over the traditional level set function. Firstly, a single fuzzy function can capture more than one phase or objects of different intensities at the same time^30,31^. Secondly, it plays an important role in efficiently imposing constraints for implementing convexity. This lead to non-dependence of the initial guess. Furthermore, for a deeper understanding of the proposed model, the mathematical analysis is presented. For the regularization of the fuzzy membership function, the Gaussian smoothing filtering is employed. Following are the major contributions of this work:

- we impose suitable removing outliers constraints supported by a well-designed theory in a variational framework for accurate image segmentation;
- we investigate a novel approach based on the power mean function equipped with a well established theoretical base;
- to guarantee unique segmentation results, a fuzzy-membership function is employed in the proposed energy functional; and
- extensive analysis on various standard data sets, it has been observed that the proposed model works well in images having multi-objects with high noise.

The rest of the paper is organized as follows. In “Related works” section, we give a brief review of related segmentation models. The design and analysis of the proposed novel model are presented in “Proposed model” section. In “Experimental results” section, a comprehensive experimental analysis is carried out both qualitative and quantitatively for types of outdoor natural, synthetic and medical images compared to existing and latest state-of-the-art segmentation techniques. Final remarks and conclusions are made in “Conclusions and future work” section.

## Related works

### Active contours without edges (CV)

To easily implement the MS model^1^, Chan *et al.* (CV)^9^ restricted the MS model to a piecewise function reconstruction (two phases). Chan *et al.*^9^ considered a piecewise constant function which divides the image into different homogeneous regions representing the foreground and background^47^. For the image **u**, the minimization energy functional is given by Eq. (3):

3 $$\begin{aligned} F^{CV}(a_1,a_2,\Gamma )&={\mu }\ length(\Gamma )\nonumber \\&\quad +\lambda _1\int _{inside(\Gamma )}|\textbf{u}(x,y)-c_1|^2dxdy \nonumber \\&\quad + \lambda _2\int _{outside(\Gamma )}|\textbf{u}(x,y)-c_2|^2dxdy, \end{aligned}$$

where $\lambda _1,\ \lambda _2,\ \mu \ge 0$ are constants which tune the weight between the smoothing and the fitting terms. $\Gamma$ is the contour, and $c_1$, $c_2$ are average intensities of given image $I_{0}(x,y)$ for foreground and background, respectively. This is a non-convex model, so consequently one need to tune several times the position of initial guess for the desired results^51^.

### Fuzzy energy-based minimization (FEBM)

Given an image, $\textbf{u}(x,y)$ in a spacial domain $\Omega$ Krinidis et al.^31^ proposed a segmentation model based on fuzzy function embedded in active contour variational framework which is mathematically illustrated using the following Eq. (4):

4 $$\begin{aligned}F(\Gamma ,c_1,c_2,v)&=\mu length(\Gamma )\nonumber \\&\quad +\eta _{1}\int _{\Omega }[\textbf{z}(x,y)]^{m}|\textbf{u}(x,y)-c_1|^{2}dx dy \nonumber \\&\quad +\eta _{2} \int _{\Omega }[1-\textbf{z}(x,y)]^{m}|\textbf{u}(x,y)-c_2|^{2}dx dy \end{aligned}$$

where the constants $c_1$, $c_2$ stand for average values inside and outside the contour $\Gamma$, respectively, *m* is the weight exponent (normally taking the value 2), $\eta _1,\eta _2>0$ and $\mu \ge 0$ are constants. The function $\textbf{z}(x,y)\in [0,1]$ is the fuzzy membership function representing the membership degree of $\textbf{u}(x,y)$ inside the $\Gamma$ and $1-\textbf{z}(x,y)$ is the membership degree of $\textbf{u}(x,y)$ outside the $\Gamma$. For a fast convergence of the minimization problem in Eq. (4) the authors use a fast algorithm as proposed by Song and Chan^32^. This model can segment images with multi-objects, different intensity variations objects, and hazy boundaries, however, it may not properly segment noisy images. The reason is that this model uses the same conventional least square objective function which fits the data to the mean value of the foreground and background.

### A convex variational level set model for image segmentation (CVMS)

**Figure 1:**
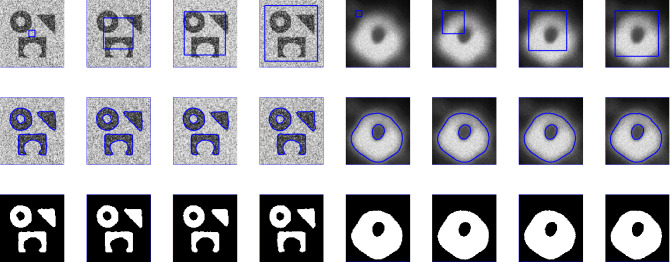
Illustration of the proposed segmentation method for the first ever black hole image and noisy images taken from^31,36^ papers. First row: different initial contours. Second row: final contours. Third row: segmented results with $p=0.5$, $\mu$  = 0.7, and $\sigma$  = 3.

**Figure 2:**
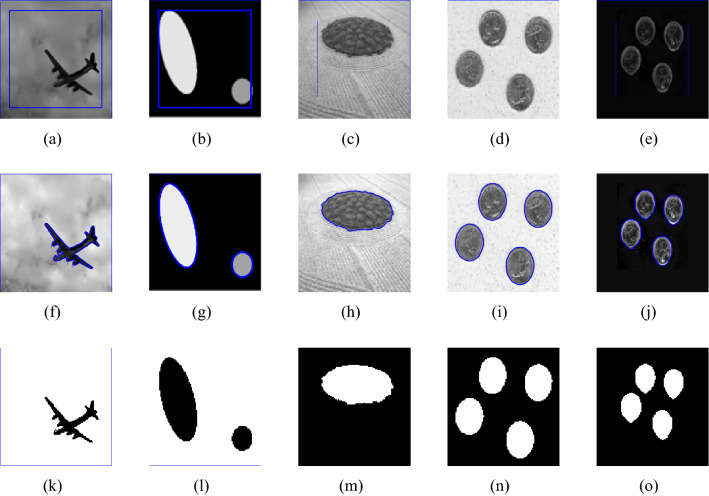
Performance of the proposed model for different images (Berkeley image data set) with $p=0.5$, $\mu$= 0.7, and $\sigma$ = 3.

**Figure 3:**
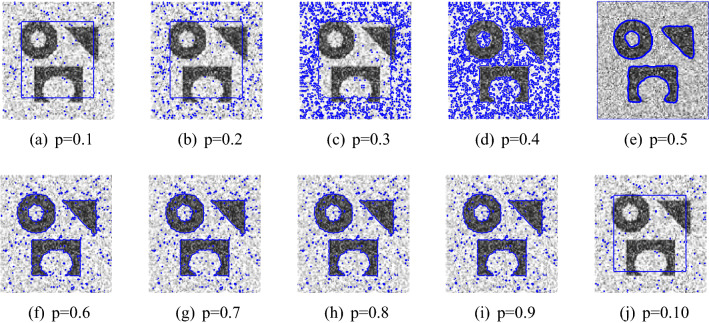
The performance of the proposed method for noisy image taken from^31,36^ papers for different values of *p*, and fixed $\mu$=0.7, $\sigma$=3, iteration$=50$.

**Figure 4:**
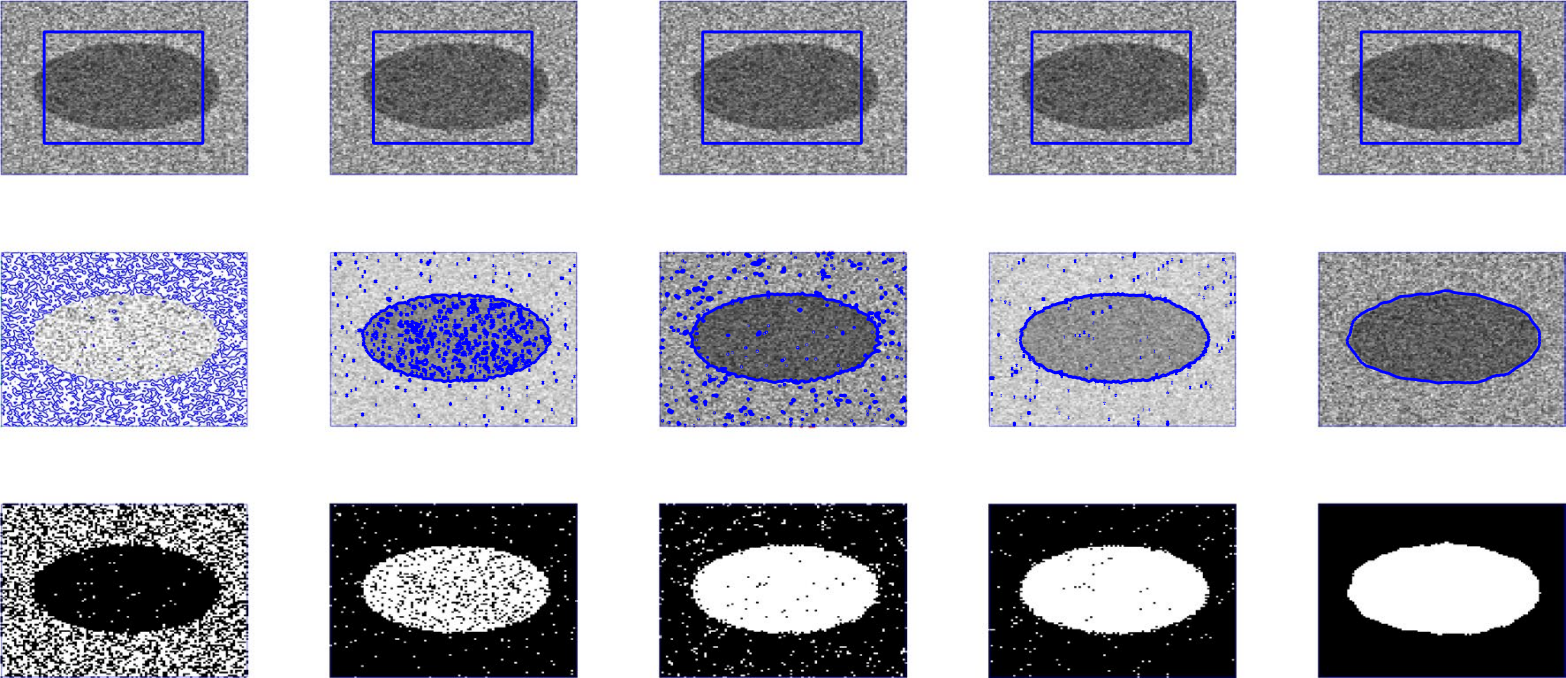
This image is taken from the Berkeley’s data set$^1$. First row shows the given image and the zero level set initialization, the second row shows the segmentation contour and the last row denotes the binary image resulting from the reconstruction of each method involved in the comparison. First, second, third and fourth columns are the segmentation results of Wu *et al.*^24^, Li *et al.*^15^, Wu *et al.*^12^ and Krinidis *et al.*^31^, respectively. The fifth column illustrates the result of proposed model with $p=0.5$, $\mu$=0.7, $\sigma$=3, with speckle noise = 0.2.

**Figure 5:**
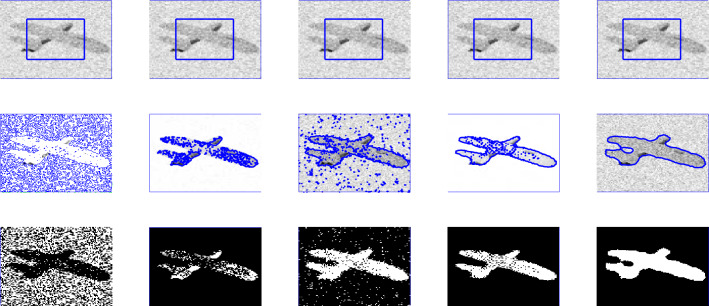
First row shows the given image and the zero level set initialization, the second row shows the segmentation contour and the last row the binary image resulting from the reconstruction of each method involved in the comparison. First, second, third and fourth columns are the segmentation results of Wu *et al.*^24^, Li *et al.*^15^, Wu *et al.*^12^ and Krinidis *et al.*^31^, respectively. The Fifth column illustrates the result of proposed model with $p=0.5$, $\mu$=0.7, $\sigma$=3, with speckle noise = 0.2.

Wu *et al.*^24^ proposed a strictly convex functional for two-phase image segmentation which is mathematically illustrated using the following Eq. (5):

5 $$\begin{aligned} F_{WH}(\psi )&=\eta \int _\Omega \frac{(\textbf{u}(x,y)-c_1)^{2}}{{c_1}^{2}}(\psi (x,y)+1)^{2}dx dy \nonumber \\&\quad +\int _\Omega \frac{(\textbf{u}(x,y)-c_2)^{2}}{{c_2}^{2}}(\psi (x,y)-1)^{2}dx dy \end{aligned}$$

where $\psi$ denotes the level set function^5^, and $\eta >0$ is a parameter. This should be noted that Eq. (5) is strictly convex, and it is flexible to its initial contour place, but it may not work for the noisy images as we can see in Figs. 4 and 5. Note that Fig. 4 has been taken from the from Berkeley’s data set and is publicly available online [https://www2.eecs.berkeley.edu/Research/Projects/CS/vision/grouping/resources.html]. In the theoretical aspect, the image data fitting term in a discrete sense is based on the concept of squared CoV, ${CoV}^2=\displaystyle {\sum _{i,j}\frac{(\textbf{u}(i,j)-\textbf{a})^2}{\textbf{a}^2}}$ whose minimum turns out to be $\textbf{a}= \frac{\displaystyle \sum _{i,j}{\textbf{u}(i,j)}^2}{\displaystyle \sum _{i,j}{\textbf{u}(i,j)}}$. The value of this average and the objective function both are sensitive to noise and outliers^27^ similar to the CV model. In fact, that is one of the main reasons that why the Wu *et al.*^12^ model is unable to work in noisy images and performs even worser than the CV model (Figs. 1, 2, 3).

### Fuzzy active contour (FAC) model

**Figure 6:**
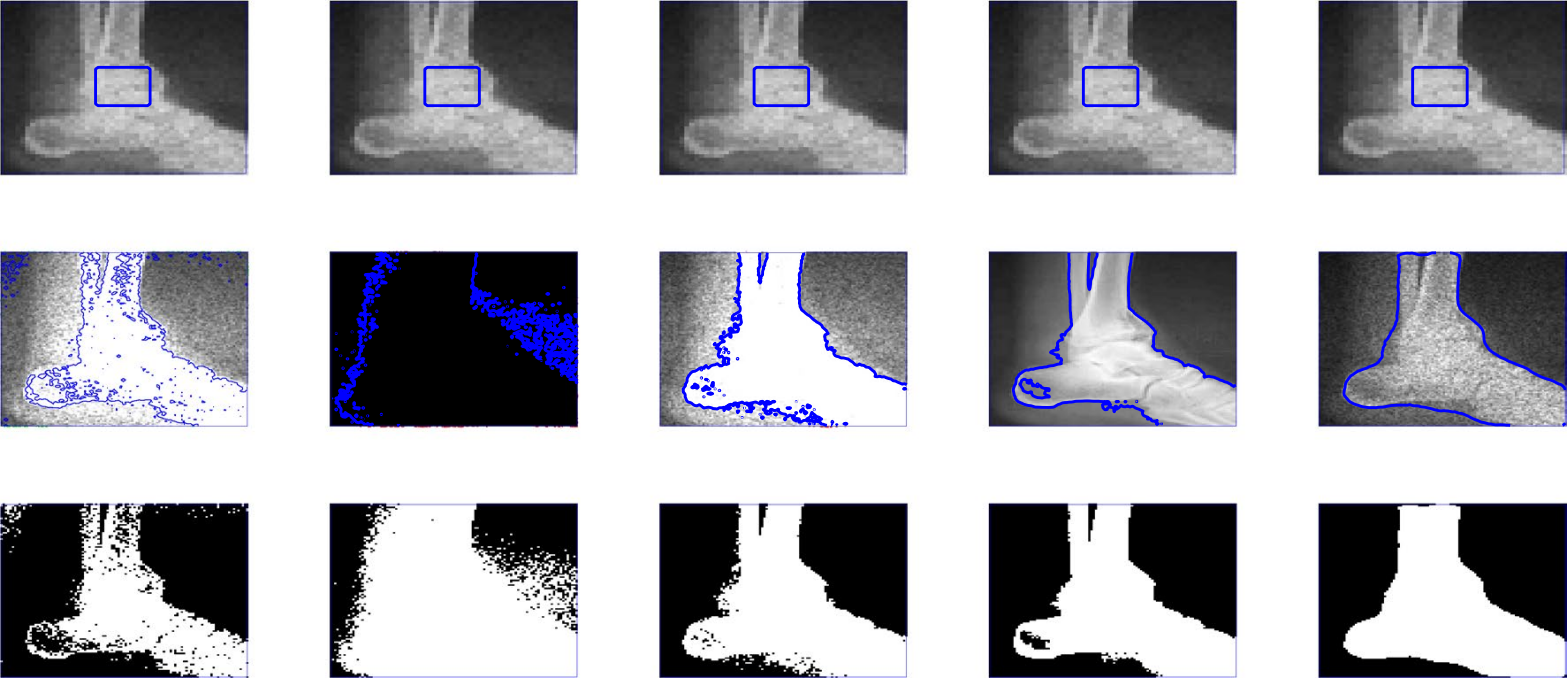
First, second, third and fourth columns are the segmentation results of Wu *et al.*^24^, Li *et al.*^15^, Wu *et al.*^12^ and Krinidis *et al.*^31^, respectively. The fifth column is the segmentation result of proposed model with p=0.6, $\mu$=0.7, $\sigma$=3, and noise = 0.1.

**Figure 7:**
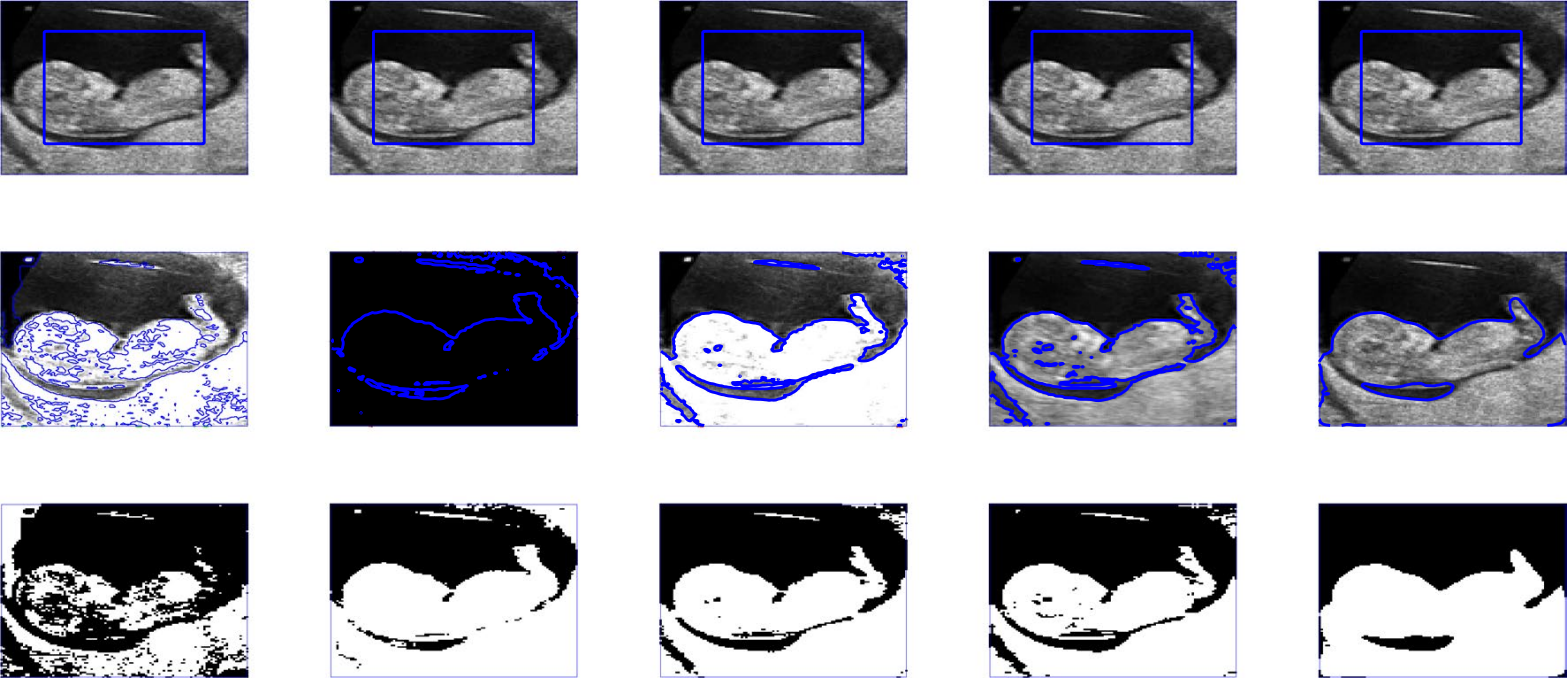
First, second, third and fourth columns are the segmentation results of Wu *et al.*^24^, Li *et al.*^15^, Wu *et al.*^12^ and Krinidis *et al.*^31^, respectively. The fifth column is the segmentation result of proposed model with $p=0.6$, $\mu$ = 0.7, and $\sigma$ = 3.

**Figure 8:**
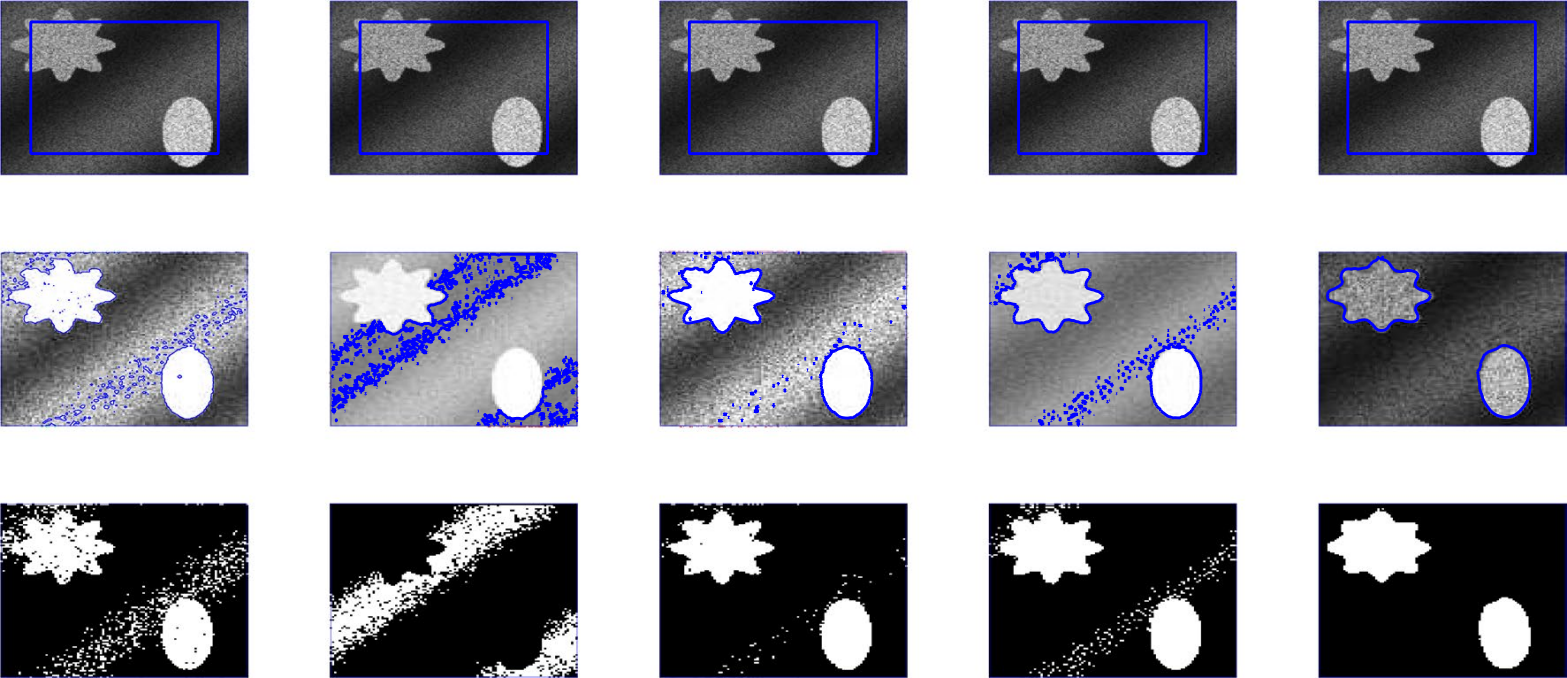
First, second, third and fourth columns are the segmentation results of Wu *et al.*^24^, Li-kim^15^, Wu *et al.*^12^ and Krinidis *et al.*^31^, respectively. The fifth column is the segmentation result of proposed model with $p=0.5$, $\mu$ = 0.7, and $\sigma$ = 3.

**Figure 9:**
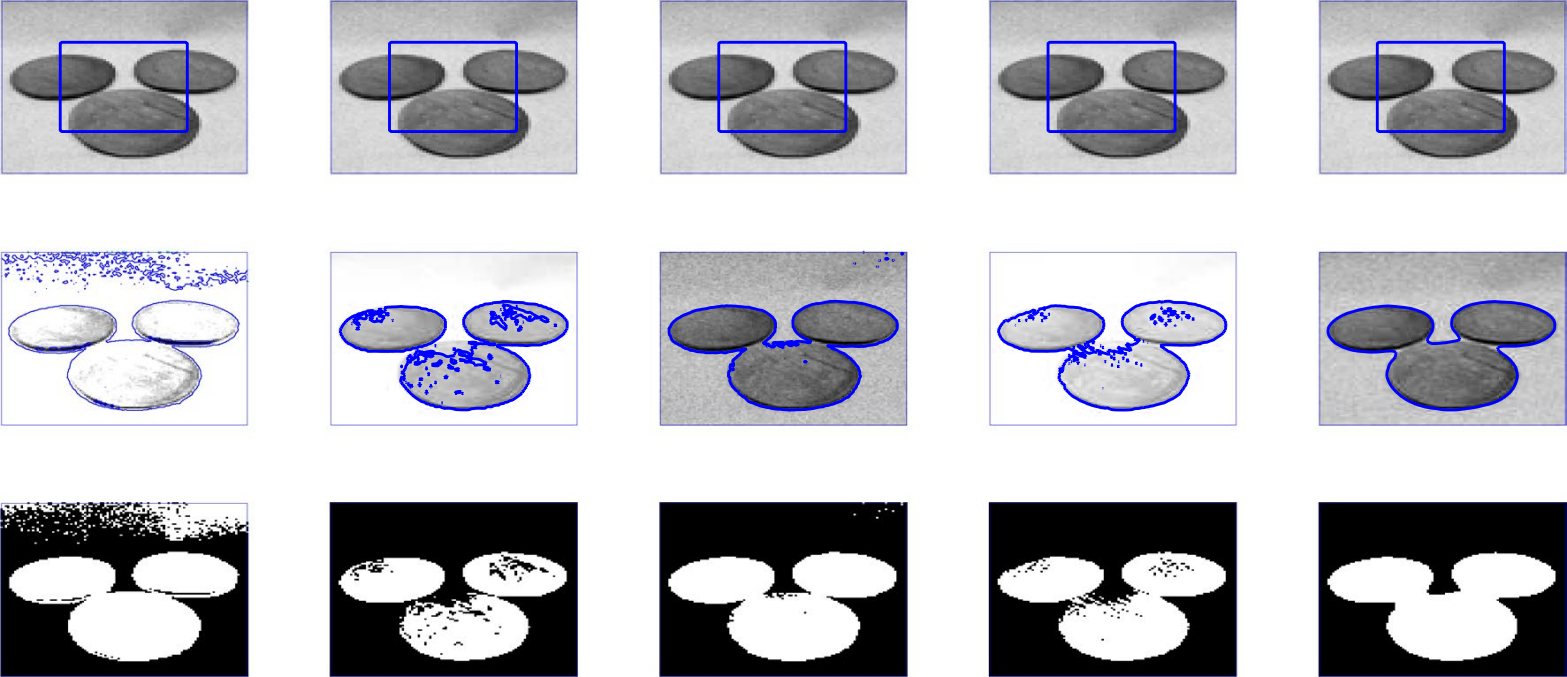
First, second, third and fourth columns are the segmentation results of Wu *et al.*^24^, Li *et al.*^15^, Wu *et al.*^12^ and Krinidis *et al.*^31^, respectively. The fifth column is the segmentation result of proposed model with $p=0.5$, $\mu$= 0.7, $\sigma$= 3, and speckle noise = 0.2.

In contrast with the traditional $L_2$ norm fidelity term based models, a fuzzy active contour model with kernel metric is proposed by Wu *et al.*^12^, which is based on the following fuzzy function given in Eq. (6):

6 $$\begin{aligned}F({\Gamma },c_1,c_2,z)&=\mu length({\Gamma }) \nonumber \\&\quad +\eta _1\int _{\Omega }[\textbf{z}(x,y)]^{m}(1-\hat{k}(\textbf{u}(x,y),c_1))dx dy \nonumber \\&\quad +\eta _2\int _{\Omega }[1-\textbf{z}(x,y)]^{m}(1-\hat{k}(\textbf{u}(x,y),c_2))dxdy, \end{aligned}$$

where the kernel metric is characterized by $\hat{k}(\xi _{1},\xi _{2})=\langle {\chi (\xi _{1}),\chi (\xi _{2})}\rangle$ and the given values for $\xi _{1}$, $\xi _{2}$ are vectors and $\chi (.)$ symbolizes a nonlinear map. Here $\langle {\chi (\xi _{1}),\chi (\xi _{2})}\rangle$ is the inner product of $\chi (\xi _{1})$ and $\chi (\xi _{2})$. Gaussian radial basis function $\hat{k}(\xi _{1},\xi _{2})$ is given by Eq. (7):

7 $$\begin{aligned} \hat{k}(\xi _{1},\xi _{2})=\exp \left( -\frac{(\xi _{1}-\xi _{2})^{2}}{\rho }\right) \end{aligned}$$

where $\rho$ is the parameter. From Figs. 4, 5, 6, 7, 8, 9 in the experimental section it is clear that Eq. (6) may not work for noisy images although $\hat{k}$ serves as a weight function which is supposed to assign suitable weights to image true pixel and outliers^48^. In the model implementation, the outliers get enough weights to affect the segmentation performance of this model in noisy images.

### Unconditional stable method for bimodal (USMB) image segmentation

Li *et al.*^15^, proposed the following energy functional which is based on Lee *et al.*^33^ idea of a stationary global minimum and is given by Eq. (8):

8 $$\begin{aligned}&F(c_1,c_2,\psi )\nonumber \\&\quad =\eta _1\int _{\Omega }(\textbf{u}(x,y)-c_1)^{2}\psi (x,y)H(1+\psi (x,y))dxdy \nonumber \\&\qquad -\eta _2\int _{\Omega }(\textbf{u}(x,y)-c_2)^{2}\psi (x,y)H(1-\psi (x,y))dxdy, \end{aligned}$$

where *H* denotes the Heaviside function and $c_1$, $c_2$ are constants.

The fact that the CV model ignores the presence of noise and other factors such as intensity in-homogeneity^23^, can be easily observed from the fitting data term used in the CV model. The fitting data term is mathematically illustrated using Eq. (9):

9 $$\begin{aligned} \displaystyle {\int _{\text {inside}(\Gamma )}|\textbf{u}(x,y)-c_1|^{2}dxdy+\int _{\text {outside}(\Gamma )}|\textbf{u}(x,y)-c_2|^{2}dxdy}, \end{aligned}$$

where $\textbf{u}(x,y)$ is the given image with $(x,y)\in \Omega$ a rectangular domain, $c_1$, $c_2$ are constants, and $\Gamma$ denotes the boundary of the objects. In discrete sense, this data term is based on the least square method and the objective function given by Eq. (10):

10 $$\begin{aligned} \displaystyle \frac{1}{\mid \Omega \mid }\displaystyle \sum _{i,j}(\textbf{u}(i,j)-{\textbf{c}})^2, \end{aligned}$$

whose minimum is the sample mean $\textbf{c}=(c_1,c_2)=\bar{\textbf{x}}$ inside and outside the $\Gamma$. From the formula, this can be easily observed that the sample mean is largely affected by the outliers.

The (LBF) model, that was anticipated by Li *et al.*, tackles intensity in-homogeneity but not noise. This phenomenon can be observed by analyzing the data fitting term as given in Eq. (11):

11 $$\begin{aligned} \displaystyle {\int _{\Omega } K_{\sigma }*|\textbf{u}(x,y)-c_1|^{2}dx dy+\int _{\Omega }K_{\sigma }*|\textbf{u}(x,y)-c_2|^{2}dx dy}, \end{aligned}$$

where $K_{\sigma }$ is Gaussian kernel. In a discrete and local sense this data term is also based on the least square method and the corresponding objective function is represented by Eq. (12):

12 $$\begin{aligned} \displaystyle \sum _{N_x}(\textbf{u}(i,j)-{{\textbf {c}}})^2, \end{aligned}$$

The above is true, particularly, in local neighborhood $N_x$ whose minimum is also the sample mean ${\textbf {c}}=\bar{\textbf{x}}$. In other words, the LBF model uses the concept of the CV model but in local neighborhoods throughout image domain $\Omega$. This leads to wider image intensity variation in small patches but on the other hand, it is more prone to noise and outliers as compared to the CV model^48–50^. In this way, the fitting term takes into account the image intensity variance in small patches but on the other hand, it is more prone to noise and outliers as compared to the CV model. Moreover, this model is not convex so consequently one need tune several times the position of initial guess for the desired results.

Li *et al.*^15^ showed that for any time step the proposed scheme is unconditionally stable. Moreover, with the assumption that $|\psi ^{n}| \le 1$ it is easy to show that $|\psi ^{n+1}| \le 1$, which leads to a straightforward update of $\psi ^{n+1}$ from given $\psi ^{n}$. Although the method shows stability for image segmentation of synthetic and real images with moderated noise the method, similar to the above ideas were the least square fit directs to the mean of the foreground and background, shows sensitivity to high noise and outliers^29^.

Ali *et al.*^34^ introduced Lehmer’s type generalized mean in an segmentation framework. Although this average is very effective in multi-region segmentation and suitable to different image intensity backgrounds it requires further analysis to tackle noise and outliers. Goldstein *et al.*^37^ used Bregman-split method which is well known for its speed, but may not work very well for images with intensity inhomogeneous. Furat *et al.*^40^ proposed techniques for the segmentation of tomographic image data of functional materials by combining machine learning methods and conventional image processing steps. This approach produced good segmentation results specially for tomographic images.

### Image segmentation with deep learning

The convolutional neural networks (CNNs) have emerged as the most popular and successful among various deep learning based models for the task of image segmentation. All of these methods are, in fact, based on the notion of machine learning techniques, and they have produced many outstanding and promising outcomes. There are additional methods that combine the concepts of CNN and active contour to solve the problem of image segmentation, such as the deep active contour network (DACN) approach developed by Zhang et al.^41^. However, the CNN method has a disadvantage in that it does a poor job of recognizing specific object boundaries. Information loss in the subsequent down sampling layers is the primary culprit^42^. The active contour models, on the other hand, produce localization of boundaries that is comparatively more precise and valuable since they fit an arch for the object form in the image using certain methods. Furat *et al.*^40^ proposed numerous techniques for the segmentation of tomographic image data of functional materials through combining machine learning methods and conventional image processing steps. With the notable exception of tomographic images, this should be noted that the model has not shown superiority for segmentation results.

Similarly, the long and short term model (LSTM) is frequently employed for image segmentation. Traditional LSTM models, however, are inadequate because they are potentially unable to extract spatial information from images. The computational costs of models may also be greatly raised by completely linked weights. Therefore, to do instance-level segmentation, convolutional LSTM approaches have essentially replaced classic LSTM models. These models have the ability to choose each instance of the item in output and sequential results with different timestamps. Due to their alleged greater control over the process of localizing specific instances than typical convolutional LSTMs, which may choose various examples of objects at different timestamps, attention models are therefore assured to further enhance the model performance. A deep learning-based denoising strategy that uses the CNN model with residual connection and attention mechanism is presented in^43^. The denoised image is produced by further removing noise once the Attention-Residual process has determined how much of it there is in the image. Other works such as^44,45^, provide an overview of several deep learning based models, including CNN, and RNN based techniques for image segmentation^46^.

## Proposed model

As mentioned above, most of the active contour region-based variational segmentation models consider ideal image while constructing the energy functional(s). This can be very easily observed by investigating the utilized image statistical information incorporated in objective functions, such as averages, the measures of dispersions, statistical variance, and standard deviation. In the literature, most of the variational region-based image segmentation models are based on the CV model fitting term idea which is sensitive to noise and outliers^29^, or similar to the works in^24,26^. Albeit, these methods are demonstrated to be very robust and effective when detecting edges and boundaries in images of low contrast; however, these methods can be very sensitive when there exists noise and outliers^27^. Therefore, other methods or, at least, improvements to the classical CV model should be made in order to ensure detection of noise and outliers in low contrast images.

To improve the state-of-the-art models mentioned in related works, we propose a new method which incorporates the power mean into the robust discrete objective function by replacing the traditional models where the arithmetic mean has been used. The sate-of-the-art with in the domain of the power mean indicates that it has the capability to discriminate the noise and intrinsic intensity^29^. To handle a noisy image one can design a formulation in the continuous framework based on averages and measure of dispersion’s. Furthermore, the employment of a fuzzy membership function has its advantages over the traditional level set function, as this allows, the involvement of less number of functions to capture many objects of different intensities^30,31^.

Initially, we discuss the power mean function and its property of canceling the negative effect of outliers. We continue in the second subsection with the presentation of the proposed model guided by a fuzzy function based formulation. The rest of the section analyses the convexity of the energy functional, its semi-continuity and coercivity.

### Power mean

#### Definition

For a given gray scale image $\textbf{u}(x,y)\in \Omega$ of size $N\times M$, power mean can be defined in discrete form as follows in Eq. (13)^29,34^:

13 $$\begin{aligned} M_{p}({\textbf {I}})=\left( \frac{1}{NM}\sum _{i=1,j=1}^{N,M}{\textbf{u}(i,j)}^{p}\right) ^{\frac{1}{p}}, \end{aligned}$$

where $p\ne 0$, and $\textbf{u}(i,j)>0$ is the intensity value at a certain pixel (*i*, *j*). For different value of *p*, such as $p=1,0,-1$, the general mean represents specific mean variations such as arithmetic, geometric or harmonic mean.

The parameter *p* controls the contribution of each sample’s element by handling each of them differently according to their significance. Oh *et al.*^29^ and Ali *et al.*^34^ has been introduced an implementation of such feature. The authors have expressed the general power mean as a linear combination of the elements in the set and its simplification form is illustrated as given in Eq. (14):

14 $$\begin{aligned}{} & {} \sum _{i=1,j=1}^{N,M}{\textbf{u}(i,j)}^{p}=\sum _{i=1,j=1}^{N,M}y(i,j)\textbf{u}(i,j), \nonumber \\{} & {} \quad y(i,j)=\textbf{u}(i,j)^{p-1}, \text { for } i=1,2,...,N\ \text {and}\ j=1,2,...,M. \end{aligned}$$

The employment of the generalized mean controls the existing trade-off between the negativeness of outliers in the observed set. It is easy to observe that, the generalized mean in Eq. (14) is an arithmetic mean if $p = 1$. The weight *y*(*i*, *j*) decreases (increases) as $\textbf{u}(i,j)$ increases (decreases) if $p\le 1$. This indicates that Eq. (14) is more affected by the small intensity values in the given image $\{\textbf{u}(i,j)\}_{i=1,j=1}^{N, M}$ and if *p* decreases, the extent of the effeteness increases. In^35^, this information played a key role in applying the generalized mean approach. To develop the ancient models, Oh *et al.*^29^ exchange the conventional least square sample mean fitting term with the generalized mean fitting term as given below in Eq. (15):

15 $$\begin{aligned} m_G({\textbf {I}})={\arg \min }_\textbf{a}\left( \frac{1}{NM}\sum _{i=1,j=1}^{N,M}\left( ||\textbf{u}(i,j)-\textbf{a}||_{2}^{2}\right) ^{p}\right) ^{\frac{1}{p}}, \end{aligned}$$

where $\textbf{a}$ is any arbitrary value in a given image intensity values. We observe that Eq. (15) converts to the traditional CV objective function for $p=1$ which is based on the conventional arithmetic mean of the squared distance^29,34^. One can choose $p < 1$^29,34^ to reduce the negative effects of outliers. In such a way, as *p* decreases the contribution of a large number to the objective function decreases. This means that the power mean can discriminate the noise and intrinsic intensity. Furthermore, Eq. (15) can be written as given by Eq. (16)^29,34^:

16 $$\begin{aligned} m_G({\textbf {I}})={\arg \min }_\textbf{a}\sum _{i=1,j=1}^{N,M}\left( ||\textbf{u}(i,j)-\textbf{a}||_{2}^{2}\right) ^{p}. \end{aligned}$$

The basic condition for the generalized sample mean $m_G$ to be a local minimum of the objective function (15) is that the gradient of this function with respect to $\textbf{a}$ is equal to zero^29,34^, that is mathematically described as given by Eq. (17).

17 $$\begin{aligned} \frac{\partial }{\partial \textbf{a}}\Big (\sum _{i=1,j=1}^{N,M}\left( ||\textbf{u}(i,j)-\textbf{a}||_{2}^{2}\right) ^{p}\Big )=0. \end{aligned}$$

Similar to the expectation-maximization algorithm scheme, Oh *et al.*^29^ developed an iterative form for easily solving Eq. (16). First, rewriting Eq. (16) in the form of Eq. (14) and then approximated by a quadratic function given by Eq. (18):

18 $$\begin{aligned} ||\textbf{u}(i,j)-\textbf{a}||_{2}^{2} \end{aligned}$$

which can be optimized as illustrated in Eq. (19):

19 $$\begin{aligned} \sum _{i=1,j=1}^{N,M}\left( ||\textbf{u}(i,j)-\textbf{a}||_{2}^{2}\right) ^{p}\approx \sum _{i=1,j=1}^{N,M}{\beta (i,j)}^{(k)}||\textbf{u}(i,j)-\textbf{a}||_{2}^{2} \end{aligned}$$

where $\beta$ is denoted using Eq. (20):

20 $$\begin{aligned} {\beta ^{(k)}(i,j)}=\left( ||\textbf{u}(i,j)-\textbf{a}^{(k)}||_{2}^{2}\right) ^{p-1}, \end{aligned}$$

for *k* number of the iterations. The approximation is exact when $\textbf{a} = \textbf{a}^{(k)}$. Here, $\textbf{a}^{(k)}$ can be updated based on the computed ${\beta (i,j)}$ in Eq. (19). The approximated function, which based on the computed value of ${\beta (i,j)}$, is mathematically expressed as given in Eq. (21):

21 $$\begin{aligned} \frac{\partial }{\partial \textbf{a}}{\beta ^{(k)}(i,j)}||\textbf{u}(i,j)-\textbf{a}||_{2}^{2}=0. \end{aligned}$$

Then, as a weighted average of the samples $\textbf{a}^{(k+1)}$ can be computed by (20) and it gives to the following Eq. (22):

22 $$\begin{aligned} \textbf{a}^{(k+1)}=\frac{1}{\sum _{i=1,j=1}^{N,M}{\beta ^{(k)}(i,j)}}\Big (\sum _{i=1,j=1}^{N,M}{\beta ^{(k)}(i,j)}\textbf{u}(i,j)\Big ). \end{aligned}$$

It is important to point out that the function $\beta$ serves as a weight function that assigns suitable weights to the true image pixels and outliers^29,34^. The parameter *p* controls the function $\beta$ and its optimal tuning value has been shown to be in the range $0.6\le p\le 0.8$^29,34^. In the following section, we show a new implementation of generalized mean in fuzzy membership variational segmentation framework, which has been fully studied in the work of Oh *et al.*^29^.

### A new fuzzy function segmentation model led by data-guided outliers avoidance

Defining the image $\textbf{u}$ on $\Omega \subset \Re ^{2}$, and $\Omega _i \subseteq \Omega$ are disjoint connected open subsets with a piecewise smooth boundary *C* ($\cup _{i}\Omega _i$). $\{C_i\in \Re ^2|^n_{i=1}\}$ are the curves of the samples to be segmented and $\{c_i\in \Re ^2|^n_{i=1}\}$ are their homogeneous associated means. The task of image segmentation is to divide an image into *n* group of data samples $\{C_i|^n_{i=1}\}$. To improve the segmentation accuracy in the presence of outliers we investigate a novel approach where the quality of generalized sample mean is taken into account and incorporated as a fitting term in a minimization functional. Concretely, we use the Euclidean distance of an input sample $C_i$ to representative samples $c_i$ by allowing in this way some pixels of $C_i$ to be recognized as outliers. In this case, the $c_i$ value not necessary must be near to these samples which consequently brings robustness to outliers. Based on this simple idea we can modify the Chan-Vese active contour model, and described it in the following form using Eq. (23):

23 $$\begin{aligned}{}  {} F(c_{1},c_{2},C)&=\mu length(C)\nonumber \\{} & {} \quad +\Big (\frac{1}{N_1(C)}\int _{inside(C)}(\Vert \textbf{u}(x,y)-c_{1}\Vert _{2}^{2}dxdy)^{p}\Big )^\frac{1}{p}\nonumber \\{} & {} \quad +\Big (\frac{1}{N_2(C)}\int _{outside(C)}(\Vert \textbf{u}(x,y)-c_{2}\Vert _{2}^{2}dxdy)^{p}\Big )^\frac{1}{p}, \end{aligned}$$

here we have three terms, first on is the curve length term, the second and third terms, which we will further refer as $$F_1(C)$$ and $$ F_2(C)$$, are the new fitting-terms with $$N_1(C)$$ and $$N_2(C)$$ the number of points inside and outside the curve *C*, respectively. By the same argument, as we get Eq. (16), the values of $$F_1(C)$$ and $$ F_2(C)$$ are computed using Eqs. (24) and (25):

24 $$\begin{aligned}{} & {} F_{1}(C)=\int _{inside(C)}(\Vert \textbf{u}(x,y)-c_{1}\Vert _{2}^{2}dxdy)^{p} \end{aligned}$$

25 $$\begin{aligned}{} & {} F_{2}(C)=\int _{outside(C)}(\Vert \textbf{u}(x,y)-c_{2}\Vert _{2}^{2}dxdy)^{p}, \end{aligned}$$

and by the same way to Eq. (19), we get this mathematical illustration and the values of $$F_1(C)$$ and $$ F_2(C)$$ are computed using Eqs. (26) and (27):

26 $$\begin{aligned}{} & {} F_{1}(C) \approx \int _{inside(C)}\alpha (x,y)\Vert \textbf{u}(x,y)-c_{1}\Vert _{2}^{2}dxdy \end{aligned}$$

27 $$\begin{aligned}{} & {} F_{2}(C) \approx \int _{outside(C)}\beta (x,y)\Vert \textbf{u}(x,y)-c_{2}\Vert _{2}^{2}dxdy, \end{aligned}$$

where

28 $$\begin{aligned} \alpha (x,y)=(\Vert \textbf{u}(x,y)-c_{1}\Vert _{2}^{2})^{p-1}, \end{aligned}$$

and

29 $$\begin{aligned} \beta (x,y)=(\Vert \textbf{u}(x,y)-c_{2}\Vert _{2}^{2})^{p-1}. \end{aligned}$$

Incorporating the fuzzy membership function $\textbf{z}(x,y)$, Eq. (16) can be rewritten as Eq. (30):

30 $$\begin{aligned} &\int _{z>0.5}\alpha (x,y)\Vert \textbf{u}(x,y)-c_{1}\Vert _{2}^{2}dxdy\nonumber \\= & {} \int _{\Omega }\alpha (x,y){\Vert \textbf{u}(x,y)-c_{1}\Vert _{2}^{2}}[\textbf{z}(x,y)]^{m}dxdy, \end{aligned}$$

and

31 $$\begin{aligned} \int _{z<0.5}{} & {} \beta (x,y)\Vert \textbf{u}(x,y)-c_{2}\Vert _{2}^{2}dxdy\nonumber \\= & {} \int _{\Omega }\beta (x,y){\Vert \textbf{u}(x,y)-c_{2}\Vert _{2}^{2}}[1-\textbf{z}(x,y)]^{m}dxdy, \end{aligned}$$

where $\alpha$, $\beta$ are updated through $c_{1}$ and $c_{2}$ in each step using Eqs. (28), (29) and $\textbf{z}$ is the fuzzy membership function.

We propose the following minimization functional which is mathematically expressed as given in Eq. (32):

32 $$\begin{aligned}F(\textbf{z},c_{1},c_{2})&= \mu \int _{\Omega }|\nabla \textbf{z}(x,y)|dxdy \nonumber \\&\quad +\int _{\Omega }\alpha (x,y){\Vert \textbf{u}(x,y)-c_{1}\Vert _{2}^{2}}[\textbf{z}(x,y)]^{m}dxdy\nonumber \\&\quad +\int _{\Omega }\beta (x,y){\Vert \textbf{u}(x,y)-c_{2}\Vert _{2}^{2}}[1-\textbf{z}(x,y)]^{m}dxdy. \end{aligned}$$

Keeping $c_{1}$ and $c_{2}$ fixed in Eq. (32), then minimizing $F(\textbf{z},c_{1},c_{2})$ with respect to $\textbf{z}$, we get the associated Euler-Lagrange equation for $\textbf{z}$, *t* is an artificial time parameterizing the descent direction as mathematically illustrated in Eq. (33):

33 $$\begin{aligned} \frac{\partial \textbf{z}}{\partial t}= & {} \mu \nabla \Big ({\nabla \textbf{z} \over |\nabla \textbf{z}|}\Big ) \nonumber \\{} & {} \quad - m\alpha (x,y) [\textbf{z}(x,y)]^{m-1}\Vert \textbf{u}(x,y)-c_{1}\Vert _{2}^{2} \nonumber \\{} & {} \quad +m\beta (x,y) [1-\textbf{z}(x,y)]^{m-1}\Vert \textbf{u}(x,y)-c_{2}\Vert _{2}^{2}=0 \nonumber \\{} & {} \quad \text{ in}\,  (0,\infty )\times \Omega , \end{aligned}$$

with

34 $$\begin{aligned}{} & {} \textbf{z}(0,x,y)=\textbf{z}(x,y) \text{ in }  \Omega \nonumber \\{} & {} \quad \frac{\textbf{z}}{|\nabla \textbf{z}|}\frac{\partial \textbf{z}}{\partial \overrightarrow{n}}  \text{ on }  \partial \Omega , \end{aligned}$$

where $\overrightarrow{n}$ is the normal to the boundary $\partial \Omega$ in exterior, $\alpha (x,y)$ is defined in Eq. (28), $\beta (x,y)$ is defined in Eq. (29) and $\frac{\partial \textbf{z}}{\partial \overrightarrow{n}}$ is the normal derivative of $\textbf{z}$ at $\partial \Omega$. It is important to note that $c_1$ and $c_2$ are updated through $\alpha$ and $\beta$ in each step using Eq. (22). This should be noted that the values for $c_1$ and $c_2$ are given by Eqs. (35) and (36), respectively.

35 $$\begin{aligned}{} & {} c^{}_{1}=\frac{1}{\int _{\Omega }\alpha ^{}(x,y)[\textbf{z}(x,y)]^{m}}\int _{\Omega }\alpha ^{}(x,y)\textbf{u}(x,y)[\textbf{z}(x,y)]^{m}, \end{aligned}$$

36 $$\begin{aligned}{} & {} c^{}_{2}=\frac{1}{\int _{\Omega }\beta ^{}(x,y)[1-\textbf{z}(x,y)]^{m}}\int _{\Omega }\beta ^{}(x,y)\textbf{u}(x,y)[1-\textbf{z}(x,y)]^{m}. \end{aligned}$$

Keeping $c_{1}$, $c_{2}$ fixed and $\mu =0$, then minimizing the energy functional (31) with respect to the fuzzy membership function $\textbf{z}$, as in^31^ we get the value of $\textbf{z}$ using Eq. (37):

37 $$\begin{aligned} z=\frac{1}{1+\left( \frac{\alpha (x,y)||\textbf{u}(x,y)-c_1||_{2}^{2}}{\beta (x,y)||\textbf{u}(x,y)-c_2||_{2}^{2}}\right) ^{\frac{1}{m-1}}}. \end{aligned}$$

Moreover, this updated value is used in the numerical explicit solution of the following Euler Lagrange’s mathematical model which is given by Eq. (38):

38 $$\begin{aligned} \frac{\partial \textbf{z}(x,y)}{\partial t}= & {} \mu \nabla \frac{\nabla \textbf{z}(x,y)}{|\nabla \textbf{z}(x,y)|}\nonumber \\{} & {} \quad + m[\textbf{z}(x,y)]^{m-1}||\textbf{u}(x,y)-c_1||_{2}^{2}\nonumber \\{} & {} \quad + m[1-\textbf{z}(x,y)]^{m-1}||\textbf{u}(x,y)-c_2||_{2}^{2}. \end{aligned}$$

With the introduction of a time step $\Delta t$, the above equation can be solved with the time marching method as given through the following Eq. (39):

39 $$\begin{aligned} z^{k+1}(x,y)= & {} z^k(x,y)+\Delta t\Big [\mu \nabla \frac{\nabla \textbf{z}(x,y)}{|\nabla \textbf{z}(x,y)|}\nonumber \\{} & {} \quad + m[\textbf{z}(x,y)]^{m-1}||\textbf{u}(x,y)-c_1||_{2}^{2}\nonumber \\{} & {} \quad + m[1-\textbf{z}(x,y)]^{m-1}||\textbf{u}(x,y)-c_2||_{2}^{2}\Big ]. \end{aligned}$$

In the following section, we explore some mathematical properties that are related to the convexity of the proposed functional measurements, as determined in Eq. (31), which are important to obtain the global minimum.

### Convexity, Lower semi-continuity and coercivity of the energy functional

#### Theorem 1

The energy functional (32) is convex. The mathematical discussion over the proof of this theorem can be found in the Appendix.

#### Proof

(6). $$\square $$

#### Theorem 2

For the energy functional Eq. (32) and for fixed $\alpha , \beta , c_{1}, c_{2}$, there exists at least one solution $\textbf{z}^{*}$ in the admissible set $\Lambda =\{\textbf{z}: \textbf{z}\in BV(\Omega ), 0\le \textbf{z} \le 1\}$. The mathematical discussion over the proof of this theorem can be found in the Appendix.

#### Proof

(5). $$\square $$

**Figure 10:**
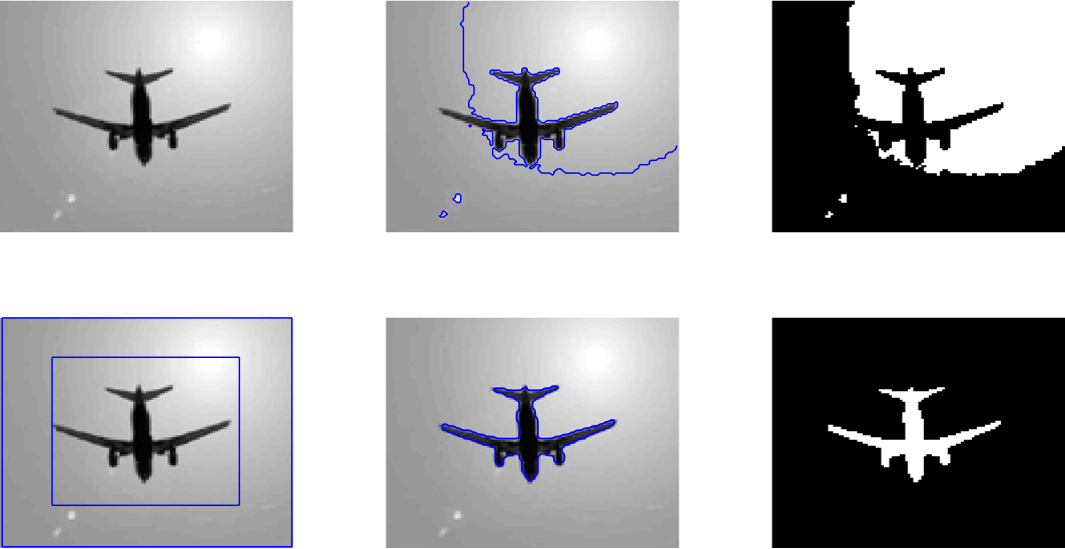
As Bregman-split method is well known for its speed. But it can be seen that the performance of our proposed model is better than Goldstein *et al.*^37^. The first row is the segmentation result of Goldstein *et al.*^37^ and second row is the segmentation result of our proposed model with $p=0.5$, $\mu$= 0.7, and $\sigma$= 0.6.

**Figure 11:**
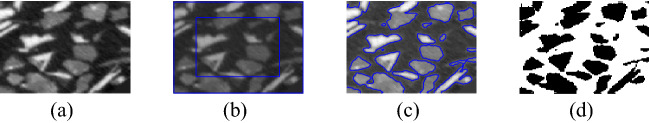
The segmentation results of the proposed model for the image taken from Furat *et al.*^40^: (**a**) 2D cut-out of tomographic image data of ore particles, (**b**) Initial contour, (**c**) Final contour and (**d**) Segmentated result of our proposed model with $p=0.6$, $\mu$= 3, and $\sigma$= 0.5.

**Figure 12:**
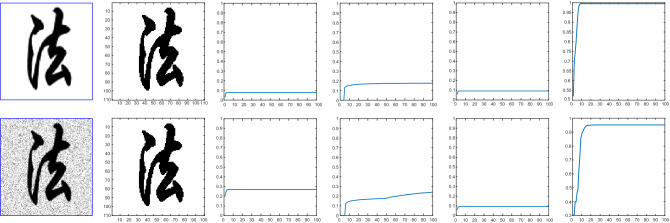
First row image is a clear image, second row denotes a nosy image. The second column shows the clean image. The Jaccard similarity coefficient for Wu *et al.*^24^, Li *et al.*^15^, Krinidis *et al.*^31^ and the proposed model with $p=0.5$,$\mu$= 0.7, and $\sigma$= 3 is shown in the third, forth, fifth, and sixth column. The x-axis denotes the iterations and y-axis is the Jaccard accuracy in each time step iteration.

**Figure 13:**
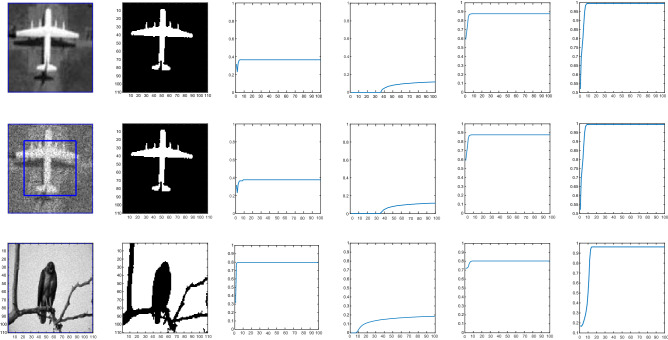
First row is a clear image, second row is noisy image, and third row is noisy and is taken from the Berkeley’s data set. The second column shows the clean image. The Jaccard similarity coefficient for Wu *et al.*^24^, Li *et al.*^15^, Krinidis *et al.*^31^ and the proposed model with $p=0.5$, $\mu =0.7$, and $\sigma$= 3 is shown in the third, forth, fifth and sixth column. The x-axis denotes the iterations and y-axis is the Jaccard accuracy in each time step iteration.

## Experimental results

In this section, we present experiments for real and synthetic image compare the performance of our method to other existing models such as Wu *et al.*^24^, Krinidis *et al.*^31^, Li *et al.*^15^, and Wu *et al.*^12^. The images used in our experiments are of a wide range including medical and real-world images having different sizes and different noise level. The proposed model is also tested for images with intensity in-homogeneity and compared with Goldstein *et al.*^37^. Moreover, different initial guesses have been applied to show the proposed model does not depend on the initialization and stuck in local minima. In our experiments the parameters $\mu =0.7$, $p=0.5$ ($p=0.6$) has been fixed through the experiments. Through the experiments, we observed that for the parameter *p* in the range of $0.5\le p \le 0.9$ the new model works, but from Fig. 3 it is clear that the best value for *p* is 0.5. All the experiments were performed on a 1.61 GHz Core $m3-7y30$ CPU @1.00 GHz with 8 GB memory. The algorithm was implemented and carried out using Matlab 9.4, in Windows 10 environment. The image size varies from $100 \times 100$ to $256 \times 256$. The datasets and images used during the experimental study are publicly available in the kaggle repository, and can be accessed at [https://www.kaggle.com/datasets/mnavaidd/image-segmentation-dataset].

### Test Set 1: Global minima achievement of the new model

To show the global minima achievement of the proposed model due to its convexity property we run experiments with diferent initialization. Figure 1 consist of two images (noisy image with three objects and first-ever black hole image) with different initial guess as shown in the first row. As clearly the proposed model does not depend on the initial guesses to archive the same segmentation results. This indicates that the method is independent on the initialization and that there is no need to check several times for different initial points. Fig. 2 shown the performance of the proposed model for different images, taken from the Berkeley image data set, with $p=0.5$, $\mu$ = 0.7, and $\sigma$=3. Figure 3 is the experimental results of our proposed model for different value of *p*, it shows that the best value for *p* is 0.5.

### Test Set 2: Robustness and accuracy of the new model

This test set consist of showing the successful performance of the proposed model on noisy images with a single and multiple objects in comparison with well-known models, such as Wu *et al.*^24^, Krinidis *et al.*^31^, Li *et al.*^15^, and Wu *et al.*^12^. Figure 4 (Berkeley’s data set) and 5 are images in presence of high noise and outlier, Figs. 6 and 7 are medical images, and Figs. 8 and 9 are noisy images with multi-objects. From all this experiments it can be observed that Wu *et al.*^24^, Krinidis *et al.*^31^, Li *et al.*^15^, and Wu *et al.*^12^ fail or partially fail to properly segment the objects in the given images whereas the proposed method gives satisfactory results.

### Test Set 3: Comparison of the proposed model on images with intensity inhomogeneity

Figure 10 shows the comparison of the proposed model and Goldstein *et al.*^37^. The images with intensity inhomogeneity are also taken from the Goldstein *et al.*^37^. The images are publicly available online [*https://sites.google.com/a/istec.net/prodrig/Home*]. This can be observed that the proposed method gives satisfactory results as compare to Goldstein *et al.*^37^. Similarly, Fig. 11 offers a comparison of obtained results using the proposed approach and the model demonstrated in^40^. Note that, the image were taken from Furat *et al.*^40^.

### Test Set 4: Accuracy analysis through Jaccard Similarity (JS) coefficient and Sørensen-Dice similarity

We evaluate the accuracy of the proposed model using the Jaccard similarity coefficient and Sørensen-Dice similarity index^38^. One can quantifying the similarities between the obtained image *X* and the ground truth *Y* using the Jaccard index that is mathematically defined by Eq. (40):

40 $$\begin{aligned} J(X,Y)=\frac{|X \cap Y|}{|X\cup Y|} \end{aligned}$$

In Figs. 12 and 13 we show the quantitative comparison of our proposed model compared to the other existing models such as Wu *et al.*^24^, Krinidis *et al.*^31^, and Li *et al.*^15^ for 5 different images with or without noise. It can be observed that Krinidis *et al.*^31^ produced relatively better results compared to Wu *et al.*^24^ and Li *et al.*^15^, but the results of the proposed model are better than Krinidis *et al.*^31^ as clearly seen in the last column of those figures. From the quantitative comparisons, it can be seen that the proposed model performs better than other existing models^15,24,31^. Table 1 shows the JS coefficients comparison of our model with other competing models. The results of this table show 10 images from Berkeley’s data set. It can be observed that in terms of accuracy the proposed model is performing better than the competing three other models in almost each image.

**Table 1:** Jaccard similarity measure, number of iterations and CPU time (second) of Krinidis *et al.*^31^, Wu *et al.*^24^, Li *et al.*^15^ and of our proposed model on 10 images from Berkeley’s data set, image size 110 × 110.

Image	Proposed model			Krinidis et al.^31^			Wu et al.^24^			Li et al.^15^		
	Iter.	JS	Time	Iter.	JS	Time	Iter.	JS	Time	Iter.	JS	Time
1	50	0.9999	0.0674	50	0.9912	0.0935	300	0.7525	0.1595	1000	0.2806	0.1085
2	100	0.8872	0.0850	100	0.8720	0.1395	300	0.7747	0.1705	1000	0.4780	0.0975
3	100	0.9210	0.0873	100	0.8890	0.1203	300	0.4976	0.1773	800	0.3003	0.0935
4	100	0.7989	0.0845	100	0.7922	0.1253	250	0.7227	0.1543	1000	0.4043	0.0971
5	100	0.7210	0.0715	100	0.6709	0.1257	300	0.5606	0.1784	900	0.5437	0.0926
6	80	0.9194	0.0656	100	0.5488	0.0936	300	0.5427	0.1475	1000	0.3563	0.0981
7	100	0.7273	0.0881	100	0.5342	0.0982	300	0.3440	0.1421	1000	0.2863	0.1103
8	100	0.7840	0..0752	100	0.7839	0.1052	300	0.7372	0.1522	1000	0.5321	0.0973
9	100	0.8458	0.0661	100	0.8325	0.1203	300	0.6735	0.1253	1000	0.3425	0.0953
10	100	0.8053	0.0771	100	0.7832	0.1283	300	0.7452	0.1523	1000	0.4731	0.1150

### Sørensen-Dice similarity

The Sørensen-Dice similarity is computed using Eq. (41):

41 $$\begin{aligned} D(X,Y)=\frac{2|X \cap Y|}{|X|+|Y|}. \end{aligned}$$

The Sørensen-Dice similarity values are normalized and given with in the range of [0, 1]. The higher Dice value shows better segmentation results and vice versa.

**Table 2:** Sørensen-Dice similarity for Krinidis *et al.*^31^, Wu *et al.*^24^, Li *et al.*^15^ and of our proposed model on 10 different images, $\mu$ = mean, $\sigma =$ SD.

Krinidis *et al.*^31^	Wu *et al.*^24^	Li *et al.*^15^	Proposed model
$\mu \pm \sigma$	$\mu \pm \sigma$	$\mu \pm \sigma$	$\mu \pm \sigma$
0.95 ${\pm }$ 0.082	0.91 ${\pm }$ 0.058	0.80 ${\pm }$ 0.069	0.98 ${\pm }$ 0.085

Table 2 shows the Sørensen-Dice coefficients for the comparison of our anticipated model with other competing models, for instance, Krinidis *et al.*^31^, Wu *et al.*^24^, and Li *et al.*^15^. These results were obtained from experiments on 10 different images that were suitable for interactive segmentation with a pre-labeled ground truth consisting of means of the labeled ground truth. It can be observed that Krinidis *et al.* model produced relatively better results as compared to Wu *et al.* and Li *et al.*, but for a high noisy or low intensity image it loses the details. From the results it is clear that the proposed model performs better than the other competing models.

## Conclusions and future work

This article mainly focuses to design an efficient image data term based on an unconventional and novel objective function - as given by Equation 2. The reason is that this metric is robust against the outliers by giving fewer weights to outliers and noise in contrast with the conventional and old objective function, given by Equation 10, which give importance to outliers. Besides this a fuzzy level set function is employed with two main benefits over the conventional level set function: capturing more than one phase or objects of different intensities plays an important role while designing a convex functional. In this way, one can impose constraints for convexity, which can be efficiently implemented, avoiding the initial guess tuning. For a deeper understanding of the properties of the proposed model, a mathematical analysis is presented and demonstrated. Moreover, the Gaussian smoothing filtering is employed for the regularization of the fuzzy membership function. Furthermore, for comprehensive analysis of the performance of the proposed model qualitative and quantitative measures are performed on various images. It has been observed that the proposed novel model performs far, and much, better than the existing and latest state-of-the-art segmentation techniques.

Selective image segmentation is one of the most important topics in medical imaging and real applications. In the future, we will work and propose a robust selective segmentation model using a dual-level set variational formulation model that should be based on the local spatial distance. A similar model should aim to segment all objects with one level set function (global) and the selected object with another level set function (local). Furthermore, the combination of marker distance function, edge detection, local spatial distance, and active contour without edges should be considered in the future. Outliers must be discovered and segregated during the denoising pre-processing or suitable limits must be put on the segmentation framework to ensure correct and the most appropriate image segmentation in the presence of noise and outliers. In the future, we will use suitable removing outliers criteria backed by a well-designed theory in a variational framework for accurate and appropriate image segmentation. Finally, as stated earlier that our current work lacks comparison with methods that are established over deep learning. Therefore, in the future we will compare our approach with other deep learning based methods.

## Data Availability

The datasets generated and/or analysed during the current study are publicly available in the kaggle repository, and can be accessed at [https://www.kaggle.com/datasets/mnavaidd/image-segmentation-dataset]. Moreover, various images used within the experimental work are publicly available online.

## Appendix

### Proof of Theorem 1

For simplicity, consider the energy functional in Eq. (32) as follow:

42 $$\begin{aligned} f(\zeta )=\mu f_{1}(\zeta )+f_{2}(\zeta )+f_{3}(\zeta ) \end{aligned}$$

where $$\zeta =(x,y)$$,

43 $$\begin{aligned}&f_{1}(\zeta )=\int _{\Omega }|\bigtriangledown \textbf{z}(\zeta )|d\zeta , \end{aligned}$$

44 $$\begin{aligned}&f_{2}(\zeta )=\int _{\Omega }\alpha (\zeta )||\textbf{u}(\zeta )-c_1||_{2}^{2}[\textbf{z}(\zeta )]^{m}d\zeta , \end{aligned}$$

45 $$\begin{aligned}&f_{3}(\zeta )=\int _{\Omega }\beta (\zeta )||\textbf{u}(\zeta )-c_2||_{2}^{2}[1-\textbf{z}(\zeta )]^{m}d\zeta . \end{aligned}$$

First of all, the domain $\Omega$ is convex, because it is a rectangle. The function $f_{1}(\zeta )$ is convex as in^25^. Consider

46 $$\begin{aligned} f_{2}(\zeta )=\int _{\Omega }\alpha (\zeta )||\textbf{u}(\zeta )-c_1||_{2}^{2}[\textbf{z}(\zeta )]^{m}d\zeta . \end{aligned}$$

Taking $F_{2}(\zeta )=\alpha (\zeta )||\textbf{u}(\zeta )-c_1||_{2}^{2}[\textbf{z}(\zeta )]^{m}$, where $F_{2}:\Omega \rightarrow R$ such that

47 $$\begin{aligned} f_{2}(\zeta )=\int _{\Omega }F_{2}(\zeta )d\zeta . \end{aligned}$$

Let ${\zeta _1=(x_{1},y_{1}), \zeta _2=(x_{2},y_{2})}\in \Omega$ and $\kappa \in [0,1]$, since $\Omega$ is convex, we can write:

48 $$\begin{aligned} \kappa \zeta _1+&(1-\kappa )\zeta _2=(\kappa (x_{1},y_{1})+(1-\kappa )(x_{2},y_{2})) \nonumber \\&=(\kappa (x_{1}-x_{2})+x_{2},\kappa (y_{1}-y_{2})+y_{2})\in \Omega . \end{aligned}$$

Taking the derivative of $F_{2}(\zeta )$ with respect to the function $\textbf{z}(\zeta )$, we get

49 $$\begin{aligned} \frac{\partial F_{2}}{\partial \textbf{z}}=m[\textbf{z}(\zeta )]^{m-1}\alpha (\zeta )||\mathbf {u(\zeta )}-c_1||_{2}^{2}. \end{aligned}$$

Differentiating again with respect to $\textbf{z}(\zeta )$, we get

50 $$\begin{aligned} \frac{\partial ^{2} F_{2}}{\partial \textbf{z}^{2}}=m(m-1)[\textbf{z}(\zeta )]^{m-2}\alpha (x,y)||\mathbf {u(\zeta )}-c_1||_{2}^{2}. \end{aligned}$$

$\frac{\partial ^{2} F_{2}}{\partial \textbf{z}^{2}} \ge 0$, as $\textbf{z}(\zeta ) \in [0,1]$, $m=2$, $\alpha (\zeta ) \ge 0$ and $||\textbf{u}-c_1||_{2}^{2} \ge 0$, also $\Omega$ is convex. Thus $F_{2}(\zeta )$ is convex and for all $\zeta _1, \zeta _2 \in \Omega$ and $\kappa \in [0,1]$ the inequality

51 $$\begin{aligned} F_{2}(\kappa \zeta _1+(1-\kappa )\zeta _2) \le \kappa F_{2}(\zeta _1)+(1-\kappa )F_{2}(\zeta _2) \end{aligned}$$

holds. From Eq. (51), we have

52 $$\begin{aligned}&\int _{\Omega }F_{2}(\kappa \zeta _1+(1-\kappa )\zeta _2)d\zeta \nonumber \\&\quad \le \kappa \int _{\Omega }F_{2}(\zeta _1)d\zeta +(1-\kappa )\int _{\Omega }F_{2}(\zeta _2)d\zeta . \end{aligned}$$

Using Eq. (42), we get

53 $$\begin{aligned} f_{2}(\kappa \zeta _1+(1-\kappa )\zeta _2) \le \kappa f_{2}(\zeta _1)+(1-\kappa )f_{2}(\zeta _2), \end{aligned}$$

which means that $f_{2}$ is convex. In the same way, one can prove the convexity of $f_{3}$. Thus, $f(\zeta )$ is convex with respect to $\textbf{z}(\zeta )$ being the sum of convex functions. $\square$

### Proof of Theorem 2

Let $\{\textbf{z}^{n}\}$ be a minimizing sequence of the energy functional Eq. (32), then there exists a constant *M*, such that $F(\textbf{z}^{n},c_1,c_2,\alpha ,\beta ) \le M$. This implies that

54 $$\begin{aligned}&\mu \int _{\Omega }|\bigtriangledown \textbf{z}^{n}(x,y)|dxdy \nonumber \\&\quad +\int _{\Omega }\alpha (x,y)||I-c_1||_{2}^{2}[\textbf{z}^{n}(x,y)]^{m}dxdy \nonumber \\&\quad + \int _{\Omega }\beta (x,y)||I-c_2||_{2}^{2}[1-\textbf{z}^{n}(x,y)]^{m}dxdy \le M. \end{aligned}$$

The constraint $0\le \textbf{z} \le 1$, ensure that $\{\textbf{z}^{n}\}$ is uniformly bounded in $BV(\Omega )$. Moreover, $BV(\Omega )$ is compact w.r.t $BV_{w}^{*}(\Omega )$ topology, then for the subsequence which we also denote by $\{\textbf{z}^{n}\}$, $\exists$ $\{\textbf{z}^{*}\} \in BV(\Omega )$ such that $\textbf{z}^{n} \xrightarrow {L^{1}(\Omega )} \textbf{z}^{*}$ and $\textbf{z}^{n} \rightarrow \textbf{z}^{*}$ a.e $x \in \Omega$ and by convergence result the constraint $0\le \textbf{z}^{*} \le 1$ also holds. Since $\textbf{z}^{n} \rightarrow \textbf{z}^{*}$ implies that $[\textbf{z}^{n}]^{m} \rightarrow [\textbf{z}^{*}]^{m}$ and this implies that $\alpha ||\textbf{u}-c_1||_{2}^{2}[\textbf{z}^{n}]^{m} \rightarrow \alpha ||\textbf{u}-c_1||_{2}^{2}[\textbf{z}^{*}]^{m}$. By Fatou’s lemma,

55 $$\begin{aligned}&\int _{\Omega } \alpha (\zeta ) ||\textbf{u}-c_1||_{2}^{2}[\textbf{z}^{*}(\zeta )]^{m} d\zeta \nonumber \\&\quad \le \lim _{n\rightarrow \infty } \inf \int _{\Omega }\alpha (\zeta ) ||\textbf{u}-c_1||_{2}^{2}[\textbf{z}^{n}(\zeta )]^{m} d\zeta \end{aligned}$$

similarly we can write

56 $$\begin{aligned}&\int _{\Omega } \beta (\zeta ) ||\textbf{u}-c_2||_{2}^{2}[1-\textbf{z}^{*}(\zeta )]^{m} d\zeta \nonumber \\&\quad \le \lim _{n\rightarrow \infty } \inf \int _{\Omega }\beta (\zeta ) ||\textbf{u}-c_2||_{2}^{2}[1-\textbf{z}^{n}(\zeta )]^{m} d\zeta \end{aligned}$$

also by lower semi-continuity of total variation, we have

57 $$\begin{aligned} \int _{\Omega } |\nabla \textbf{z}^{*}(\zeta )| d\zeta \le \lim _{n\rightarrow \infty } \inf \int _{\Omega } |\textbf{z}^{n}(\zeta )| d\zeta . \end{aligned}$$

From Eqs. (44), (45) and (50), we have

58 $$\begin{aligned} F(\textbf{z}^{*},c_1,c_2,\alpha ,\beta ) \le \lim _{n\rightarrow \infty } \inf F(\textbf{z}^{n},c_1,c_2,\alpha ,\beta ) \end{aligned}$$

thus $\textbf{z}^{*} \in \Lambda$ (this complete the proof).

Therefore, the minimizer of our proposed model has a global minimum. $\square$
